# Influence of breed and environment on leukocyte telomere length in cattle

**DOI:** 10.18699/vjgb-24-23

**Published:** 2024-04

**Authors:** N.S. Yudin, A.V. Igoshin, G.A. Romashov, A.A. Martynov, D.M. Larkin

**Affiliations:** Institute of Cytology and Genetics of the Siberian Branch of the Russian Academy of Sciences, Novosibirsk, Russia; Institute of Cytology and Genetics of the Siberian Branch of the Russian Academy of Sciences, Novosibirsk, Russia; Institute of Cytology and Genetics of the Siberian Branch of the Russian Academy of Sciences, Novosibirsk, Russia; Arctic State Agrotechnological University, Yakutsk, Republic of Sakha (Yakutia), Russia; Royal Veterinary College, University of London, London United Kingdom

**Keywords:** longevity, selection, cattle, breed, dairy, beef, environment, cold climate, leukocyte telomere length, долголетие, селекция, крупный рогатый скот, молочный, мясной, холодный климат, длина теломер лейкоцитов

## Abstract

High milk yield is associated with reduced longevity in high-producing dairy cattle breeds. Pre-term culling leads to high replacement heifer demand and economic losses for the dairy industry. Selection for this trait is limited because of low heritability and difficulties in phenotype measurement. Telomeres are elements found at the ends of chromosomes, consisting of repetitive DNA sequences, several thousand base pairs in length, coupled with nucleoprotein complexes. Eventually, in humans and most other animals, telomere length reduces with age. When telomeric DNA is truncated to a critical length, cell ageing, cell cycle arrest, and apoptosis are induced. As a result, telomere length can be considered as a predictor of health risks and an individual’s lifespan. The leukocyte telomere length may be used as a proxy phenotype of productive lifespan to improve cattle selection. Our objectives were to assess the effects of breed and breed group (dairy vs. beef) on the leukocyte telomere length and to estimate the effect of cold climate on this trait in Kalmyk cattle populations from the South (Rostov Oblast) and Far North (Republic of Sakha) regions of Russia. The leukocyte telomere lengths were estimated computationally from whole-genome resequencing data. We leveraged data on leukocyte telomere length, sex, and age of 239 animals from 17 cattle breeds. The breed factor had a significant effect on leukocyte telomere length across our sample. There was no difference in leukocyte telomere length between dairy and beef groups. The population factor had a significant effect on leukocyte telomere length in Kalmyk animals. In conclusion, we found that breed, but not breed group (dairy vs. beef), was significantly associated with leukocyte telomere length in cattle. Residence in colder climates was associated with longer leukocyte telomere length in Kalmyk breed cattle.

## Abstract (ru)

Высокие удои молока сопряжены с сокращением продолжительности жизни у высокопродуктивных молочных пород скота. Преждевременная выбраковка приводит к значительным экономическим потерям в молочном животноводстве и увеличению потребности в ремонтных телках. Отбор по этому признаку затруднен из-за низкой наследуемости и сложности измерения данного фенотипа. Теломеры – это структуры, находящиеся на концах хромосом, состоящие из повторяющихся последовательностей ДНК длиной в несколько тысяч пар оснований, связанных с нуклеопротеиновыми комплексами. У людей и большинства других животных длина теломер уменьшается с возрастом. Когда теломерная ДНК сокращается до критической длины, индуцируются процессы старения клеток, остановки клеточного цикла и апоптоза. В результате длину теломер можно рассматривать как предиктор рисков для здоровья и продолжительности жизни индивида. Длина теломер лейкоцитов может быть использована в качестве суррогатного фенотипа для признака продуктивного долголетия для улучшения селекции крупного рогатого скота. Целью нашей работы было – оценить влияние породы и направления продуктивности (молочное или мясное) на длину теломер лейкоцитов, а также проанализировать влияние холодного климата на этот признак в популяциях крупного рогатого скота калмыцкой породы на Юге (Ростовская область) и Крайнем Севере (Республика Саха) России. Измерение длины теломер лейкоцитов осуществлено с помощью компьютерных методов на основе данных полногеномного ресеквенирования. Мы использовали данные о длине теломер лейкоцитов, половой принадлежности и возрасте 239 животных, относящихся к 17 породам крупного рогатого скота. Фактор породы оказывает существенное влияние на длину теломер лейкоцитов в нашей выборке. Достоверных различий в длине теломер лейкоцитов между молочными и мясными группами нами не выявлено. Значительное влияние на длину теломер лейкоцитов у животных калмыцкой породы оказывает фактор популяции. Таким образом, мы обнаружили, что именно порода, но не направление продуктивности (молочное или мясное), достоверно влияла на длину теломер лейкоцитов у крупного рогатого скота. Разведение в более холодном климате было ассоциировано с большей длиной теломер лейкоцитов у крупного рогатого скота калмыцкой породы

## Introduction

High milk production correlates with poor longevity in highproducing cattle breeds, primarily Holstein Friesian (Hu et al., 2021). Pre-term culling leads to high replacement heifer demand and economic losses for the dairy industry (Grandl et al., 2019). There is a need to improve the longevity traits of dairy cattle. However, selection for these traits is limited because of low heritability and difficulties in phenotype measurement (Zhang H. et al., 2021).

Telomeres are elements found at the ends of chromosomes, consisting of repetitive DNA sequences, several thousand base pairs in length, coupled with nucleoprotein complexes (Jenner et al., 2022). They protect the chromosomes from degradation and inhibit aberrant rearrangements during cell division (Monaghan, Ozanne, 2018). Telomeres become shorter with every cell division due to the end replication problem (Chakravarti et al., 2021) but can be maintained through telomerase activity (Schrumpfová, Fajkus, 2020) and shelterin complex (de Lange, 2018). Eventually, in humans and most other animals, telomere length reduces with age (Blackburn et al., 2015; Whittemore et al., 2019). When telomeric DNA is truncated to a critical length, cell ageing, cell cycle arrest, and apoptosis are induced (Chakravarti et al., 2021; López-Otín et al., 2023). As a result, telomere length can be considered as a predictor of health risks and an individual’s lifespan. Telomere length is correlated with many age-related conditions in humans (Armanios, 2022; Rossiello et al., 2022) and reduced life expectancy in humans and other species (Wilbourn et al., 2018; Liu et al., 2019; Crocco et al., 2021)

Telomeres in cattle shorten with age, similar to most other animals (Miyashita et al., 2002). Adult Holstein dams with short telomeres are more likely to be culled than dams with long telomeres (Brown et al., 2012). Productive lifespan in Holsteins was correlated with telomere length at birth (Ilska- Warner et al., 2019), at the age of one year (Seeker et al., 2018a), as well as telomere attrition rate (Seeker et al., 2021). The telomere length in the Agerolese breed, having a long lifespan, was significantly higher than that in the Holstein breed of the same age (Iannuzzi et al., 2022). Therefore, telomere length may be used as a proxy trait of lifespan and health to improve cattle selection.

Telomere length in cattle is a complex trait controlled by both genetics and environment. However, it is still unclear to what extent these factors influence this trait. A recent metaanalysis of the heritability of telomere length showed a moderate mean heritability of this trait (0.45) in 18 vertebrate species (Chik et al., 2022). Estimates of the heritability of telomere length, even in a single species (human), may range from 0.36 (Andrew et al., 2006) to 0.70 (Broer et al., 2013). This variability in estimates appears to be due to different research methodologies. For example, in most studies, parents and offspring are of different ages. In statistical analyses, age is counted as a covariate, but this implies a linear relationship between telomere length and age, which is not always the case (Dugdale, Richardson, 2018).

The influence of environmental factors on telomere length has been well studied in human epidemiological studies. For example, a negative correlation was found between telomere length and emotional stress (Law et al., 2016), Western pattern diet (Rafie et al., 2017), cigarette smoking (Astuti et al., 2017), and environmental chemicals (Zhang X. et al., 2013). In birds, short telomeres or a high rate of telomere shortening have been associated with malaria infection (Asghar et al., 2016), increased brood size (Reichert et al., 2014), early postnatal stress (Herborn et al., 2014) and sibling competition (Mizutani et al., 2016).

Heritability estimates of leukocyte telomere length in Holstein cattle ranged from 0.32 to 0.47 (Seeker et al., 2018a, b). Fourteen candidate genes at birth and nine at first lactation were associated with this trait in this breed using a genomewide association study (Ilska-Warner et al., 2019). Our genome- wide association study of seventeen cattle breeds revealed several SNPs associated with bovine telomere length. We also confirmed the effects of loci reported by previous studies (Igoshin et al., 2023). Mastitis (Ilska-Warner et al., 2019), bovine leukaemia virus infection (Szczotka et al., 2019), oxidative stress (Ribas-Maynou et al., 2022), parturition and raising the first calf (O’Daniel et al., 2023), lameness (Ilska-Warner et al., 2019), and lactation (Laubenthal et al., 2016) have been found to be associated with cattle telomere length. The management of the farm and genetics are herdrelated factors that can significantly affect telomere length (Brown et al., 2012). However, the question remains unanswered: to what extent is telomere length in cattle determined by breed and influenced by environmental stressors, such as weather conditions?

There are two studies on the association of the animal breed and telomere length in cattle (Tilesi et al., 2010; Iannuzzi et al., 2022). Both studies found differences in telomere lengths between the two cattle breeds in the same tissues. However, it is unclear how widespread this phenomenon is across mul-tiple cattle breeds with different phylogenetic origins and ecogeographic breeding conditions. P. Kordowitzki et al. hypothesized that severely disturbed energy balance in highproducing dairy cows eventually leads to decreased regenerative capacity and premature senescence, which can beassessed by telomere length (Kordowitzki et al., 2021). The fraction of short telomeres in PBMCs of the high-producing Holstein-Friesian breed was higher than in the dual-purpose Polish Red breed, but this observation was not supported by a statistical test significance. Therefore, it remains unclear whether different breeds of cattle (dairy vs. beef) exhibit variation in telomere length

Seeker et al. suggested that heat may be an environmental stressor capable of causing telomere attrition (Seeker et al., 2021). They found a strong correlation between maximum summer temperature and telomere attrition in Holstein- Friesian cattle. Heat stress during gestation also affected the telomere length in newborn Holstein calves. A higher median temperature-humidity index during gestation resulted in calves born with shorter telomere lengths (Meesters et al., 2023). There are, however, no studies that investigate the influence of cold weather on telomere length in cattle. A single human study showed that prenatal temperature exposure below 5 °C was associated with longer telomere length in newborn babies (Martens et al., 2019).

There are only two native beef cattle breeds in Russia: Kalmyk and its derivative Kazakh Whiteheaded breeds. It is believed that the Kalmyk cattle originated in Northwest China (Dzungaria) and was brought to Russia, to the Volga area, by migrating nomadic tribes in the seventeenth century (Dmitriev, Ernst, 1989). The Kalmyk breed was created under harsh conditions: the icy wind in winter or the hot sun in summer, frequent epizootics, etc. The specific traits of the Kalmyk breed include high viability, adaptation to the harsh climate, resistance to infections, long lifespan, thickening of the epidermis at the expense of the dermis in winter, abundance of sebaceous and sweat glands in the skin compared to other breeds (Dmitriev, Ernst, 1989). In Russia, the Kalmyk beef herd is mainly found in two regions: the Republic of Kalmykia and Rostov Oblast (Kayumov et al., 2014). This breed has also been reared in the Republic of Sakha (Yakutia) since 2013 when about 200 Kalmyk cattle animals were imported from the Republic of Kalmykia (Sleptsov, Machakhtyrova, 2019). Yakutia has an extreme and severe climate, with the average winter temperature below −35 °C.

The objectives of our study were (1) to assess the effects of breed and breed group (dairy vs. beef) on the leukocyte telomere length in the sample of 239 animals from 17 cattle breeds and (2) to estimate the effect of cold climate on this trait in Kalmyk cattle populations from the South and Far North regions of Russia. We hypothesized that high milk yield or extreme cold weather may be stress factors that may have led to a change in telomere length in cattle.

## Materials and methods

Samples. In this work, we leveraged data on leukocyte telomere length, sex, and age of 239 animals from cattle breeds used in our previous study (Igoshin et al., 2023). The leukocyte telomere lengths have been estimated computationally from whole-genome resequencing data using TelSeq software (Ding et al., 2014), which is a frequently used program for this purpose and which has been confirmed by multiple experimental techniques (Ding et al., 2014; Cook et al., 2016; Pinese et al., 2020; Taub et al., 2022; Zhang D. et al., 2022). The details of the estimation procedure can be found in our previous work (Igoshin et al., 2023).

The breeds investigated are dairy (Russian Black Pied, Holstein, Kholmogory, Red Steppe, Yaroslavl), beef (Charolais, Hereford, Kalmyk, Kazakh Whiteheaded, Wagyu), and dual-purpose (Alatau, Bestuzhev, Buryat, Kostroma, Tagil, Ukrainian Grey, Yakut) (Dunin, Dankvert, 2013; Lhasaranov, 2020). Among 30 individuals of the Kalmyk cattle breed (Supplementary Material 1)1, one group of animals (n = 10) was reared in Rostov Oblast (Mechetny settlement), while the other (n = 20) was from the Republic of Sakha (Kyuyorelyakh settlement). The climatic conditions in these locations differ substantially (see the Table). All the Kalmyk animals from both locations were raised in a stall-pasture system.


Supplementary Materials are available in the online version of the paper: https://vavilovj-icg.ru/download/pict-2024-28/appx10.pdf


Population structure. Even in the absence of selection, a founder effect or, more broadly, genetic drift could lead to genetic differentiation between two isolated populations of common origin. To ensure that two populations of the Kalmyk breed are genetically indistinguishable, we performed the principal component analysis using PLINK v.1.9 (Purcell et al., 2007) (--pca option) and the analysis of population structure using fastSTRUCTURE v1.0 (Raj et al., 2014). The fastSTRUCTURE program was run with K ranging from K = 2 to K = 8. The resulting cluster memberships were visualized with PONG v.1.5 software (Behr et al., 2016). For both methods, we used an LD-pruned (PLINK: --indep-pairwise 5000 100 0.1) dataset containing genotypes of 20,184 SNPs in 116 animals (Kalmyk animals and individuals having SRA ID from Supplementary Material 1).

Statistical analysis. Like in many other studies, the distribution of LTL in our work was skewed. If not corrected, this violates the assumptions of parametric tests, thus affecting statistical power (Lantz, 2013). Therefore, associations with LTL were tested by using log-transformed LTL values (e. g. Leung et al., 2014; Lynch et al., 2016). To find out whether a breed factor contributes to LTL variation in cattle, we used ANOVA (“aov” R function) with log-transformed LTLs (log10(LTLs)) as a response variable and breed as a factor variable, accounting for age and sex: aov(logLTL ~ Age + Sex + Breed). To find out which breeds significantly differ from each other, we additionally performed a standard ANOVA post hoc test – Tukey’s HSD test utilising the “glht” function from the “multcomp” R package (Hothorn et al., 2008). Also, we combined beef and dairy breeds into two groups and tested for a difference between them: aov(logLTL ~ Age + Sex + Group). As all the Kalmyk individuals used were dams, the test for differences between this breed’s populations was conducted by accounting only for age: aov(logLTL ~ Age + Population).

For statistically significant variables we estimated the variance explained (η2) using the “eta_squared” function from the “effectsize” R package (Ben-Shachar et al., 2020) with the “partial = TRUE” option.

Preparing data for visualization and descriptive statistics. As confirmed in our study, telomere length typically decreases with age (Spearman’s ρ = –0.305, p = 1.58 × 10–6 for raw and log-transformed LTLs) (Supplementary Material 2). Therefore, for boxplot visualization and descriptive statistics, we calculated the log10(LTL) values expected given the constant age. For this purpose, we fitted a regression model (“lm” R function) with logLTL as the response variable, and age, sex (coded by 0/1) and breed (16 covariates coded by 0/1) variables as predictors. As a result, we obtained regression parameters (intercept and slopes for each predictor) and residuals. For each animal, we summed up: the intercept, the animal’s residual, and products of each predictor value and its respective slope. For the age predictor, however, actual values were substituted by the average value of 4.5 years. The resulting values represent the expectation for logLTL given the constant age of 4.5 years. These age-adjusted log10(LTLs) and their corresponding values in kilobases (hereafter “ageadjusted LTLs”) are shown in Supplementary Material 1. The descriptive statistics for breeds can be found in Supplementary Material 3.

## Results

The statistical analysis shows that breed factor has a significant ( p = 6.12 × 10–15, η2 = 0.37) effect on leukocyte telomere length across our sample of 239 individuals (see Figure, a). Tukey’s HSD test showed significant differences for 28 breed pairs (Supplementary Material 4). At the same time, there is no significant difference in LTL between dairy and beef groups ( p = 0.0748) (see Figure, b).

The statistical testing performed for the Kalmyk breed shows that the population factor has a significant ( p = 0.0283, η2 = 0.17) effect on LTL in studied Kalmyk animals, with the individuals from Rostov Oblast having shorter telomeres (see Figure, c). The results of the principal component analysis show that the Rostov and Sakha populations form two highly overlapping clusters (Supplementary Material 5). Also, fastSTRUCTURE results suggest that the two Kalmyk populations are homogeneous and possible genetic differences between them do not exceed the level of variation within other breeds (Supplementary Material 6). Therefore, the LTL differences between the two groups are most likely explained by environmental conditions

It should also be mentioned that the age variable significantly affects LTL in all tests: p = 1.46 × 10–6, η2 = 0.10 (testing for the effect of breed); p = 0.0248, η2 = 0.07 (dairy vs. beef group); and p = 0.0038, η2 = 0.27 (Kalmyk Sakha vs. Kalmyk Rostov). However, the sex factor has no significant effect on LTL either in the test for breed factor ( p = 0.205) or in the test for the factor of breed group ( p = 0.8752).

## Discussion

The primary aim of this study was to investigate the correlation between the breed type and leucocyte telomere length (LTL) in cattle. Our additional goal was to check the effect of the environment (e. g., colder climate) on the LTL within populations of the same breed grown in different climates. Based on the resequencing data of 17 cattle breeds, our findings indicate that the breed factor has a significant impact on LTL. These results align with earlier studies that reported LTL differences in pairwise comparisons between cattle breeds (Tilesi et al., 2010; Iannuzzi et al., 2022). We also found evidence for an association between LTL and differences in climatic conditions for a single cattle breed reared in different regions of Russia.

It was shown in our analysis that the breed factor contributes more to total LTL variation compared to age. The possible practical implication for this could be the use of breeds characterised by long telomeres in crossbreeding programs aimed to improve telomere-length-associated phenotypes in cattle.

Apart from cattle, studies are reporting LTL differences between Caenorhabditis elegans strains (Cook et al., 2016), outbred populations and inbred strains of mice (Mus musculus and Peromyscus leucopus) (Manning et al., 2002), and dog breeds (Fick et al., 2012). These reports suggest the existence of a genetic basis for such variability. Based on heritability estimates for LTL in Holsteins (0.32–0.47) (Seeker et al., 2018a, b), we propose that genetic factors may largely explain inter-breed differences observed in our study

Herein we compared the LTL in dairy and beef breeds. The results However did not reveal any statistically significant difference between these two groups. This finding is consistent with a previous study that compared LTLs between dairy and dual-purpose cattle breeds, which also showed no difference (Kordowitzki et al., 2021). Our study focused on distinct groups of cattle breeds, specifically dairy and beef, covering a wider range of genetics than the previous study. Also, each production breed type was represented by five breeds. Therefore, the results reported herein could provide stronger support for the lack of LTL differences between the cattle breed types. Our results are also consistent with similar studies done in other domestic species, e. g., dogs, where no difference in LTL was reported for breed groups (working, herding, hunting) (Fick et al., 2012). It appears that complementary contributions of many factors affecting a particular breed (e. g. genetic makeup, management practices, veterinary care, climate conditions, etc.) have a greater influence on the LTL than physiological features associated with different production types. This result suggests that the selection for telomere-length-associated traits will probably not lead to substantial changes in milk or meat yields.

To investigate the possible effects of environments on telomere lengths of the same cattle breed, we compared the LTLs between two populations of the Kalmyk cattle reared in different climatic conditions. We observed a significant difference in agreement with a previous human study showing an association between prenatal cold exposure and longer blood telomere length in newborns (Martens et al., 2019). Indeed, longer telomere lengths detected in animals from the Sakha Republic with colder climates compared to the control population from Rostov Oblast imply that there could be a mechanism of telomere maintenance in colder climates in cattle. In ectotherms, however, there are reports that at cooler conditions telomere shortening happens during development (Friesen et al., 2022; Burraco et al., 2023), but other authors did not confirm this observation (McLennan et al., 2018).

In bat species, Myotis myotis, average and minimum temperatures, rainfall and wind speed during the spring when bats emerge from hibernation, give birth and rear young were associated with higher telomere attrition (Foley et al., 2020). The authors, however, did not report which variable is the driver for telomere length change. The comparison of telomere length between two species of rodents hibernating at either 3 or 14 °C revealed that individuals hibernating at the warmer temperature had longer telomeres than individuals hibernating at the colder temperature (Nowack et al., 2019). The authors hypothesized that the observed effect was not related to cold climate, but rather was associated with restoration of telomere length during frequent arousals when the body temperature returns to normal values.

The mechanisms by which cold climate impacts leukocyte telomere length in cattle remain unclear. On the one hand, cold exposure may inhibit telomere shortening, since low temperature reduces the rate of cell proliferation in mammalian cells (Kanagawa et al., 2006; Fulbert et al., 2019). On the other hand, cold exposure may induce telomere elongation by influencing the components of the telomerase complex. It was shown that cold-inducible RNA-binding protein (CIRP) was essential for telomere maintenance at hypothermia conditions in vitro by regulating both reverse transcriptase TERT and the RNA subunit TERC in the telomerase core complex (Zhang Y. et al., 2016). The transcription of telomeric repeat-containing RNAs (TERRAs), which are associated with telomere stability, was induced in mice exposed to cold (Galigniana et al., 2020).

One could ask if there is an optimal ambient temperature at which the LTL in cattle would be the longest. The few studies on the effect of ambient temperature on the LTL of endothermic mammals do not allow us to answer this question unambiguously. Extremely low or high ambient temperatures lead to hypo- and hyperthermia when the body temperature deviates substantially below and above the narrow limits of the regulated range, i. e., cause stress. The influence of a large number of stressors, including extreme environmental factors, is known to be associated with shorter telomeres or an increased rate of telomere shortening (Chatelain et al., 2020; Lin, Epel, 2022).

Indirect information on the effect of moderate cold on LTL, when body temperature remains within the normal range, could be obtained from studies of the effect of body temperature on the ageing process. On the one hand, in endotherms, a small decrease in body temperature is associated with an increase in life expectancy (Conti et al., 2006; Carrillo, Flouris, 2011). On the other hand, in some cases, there is an inverse relationship (Zhao et al., 2022). For example, human females tend to have a longer life expectancy than males, but their body temperature is higher (Waalen, Buxbaum, 2011). The mechanisms that control the relationship between body temperature and life expectancy involve not only a decrease in metabolic rate when the temperature declines, but also neuroendocrine processes that indirectly affect a variety of physiological responses when temperature changes. Therefore, the optimal limits of ambient temperature at which the LTL in cattle will be longest could exist, but this question requires further study.

**Fig. 1.**
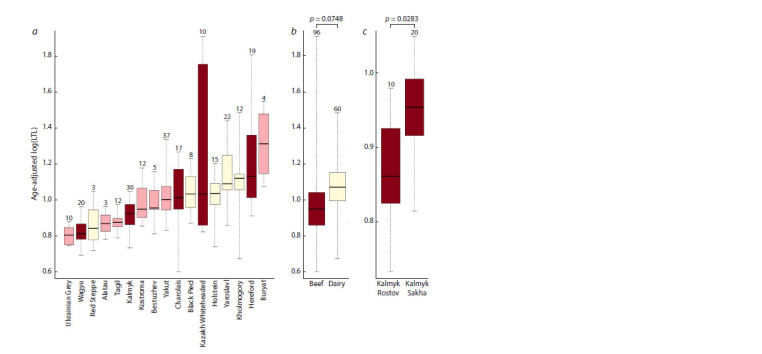
Boxplots illustrating the log-transformed and adjusted for age (expectation at 4.5 years) leukocyte telomere lengths in a) different breeds; b) beef and dairy breed groups, and c) in two populations of the Kalmyk breed. The p-values designate the statistical significance for differences between the abovementioned categories. The brown, pink and light-yellow colours correspond to beef, dual-purpose, and dairy breeds. The numbers at the top of boxplots indicate the number of animals.

**Table 1.**
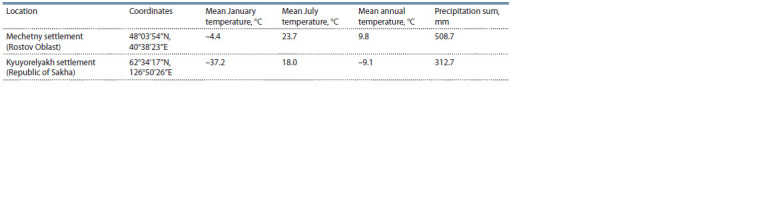
The climatic conditions for the sampling locations in Rostov Oblast and the Republic of Sakha (according to https://climatecharts.net (Zepner et al., 2021), accessed on 30 August 2023)

## Conclusion

In conclusion, we found that breed, but not breed group (dairy vs. beef ), significantly influenced leukocyte telomere length in cattle. Residence in colder climates was associated with longer leukocyte telomere length in the Kalmyk cattle breed. Our results add to the evidence regarding the influence of breed origin and cold climate on this trait in farm animals

## Conflict of interest

The authors declare no conflict of interest.

## References

Andrew T., Aviv A., Falchi M., Surdulescu G.L., Gardner J.P., Lu X.,
Kimura M., Kato B.S., Valdes A.M., Spector T.D. Mapping genetic
loci that determine leukocyte telomere length in a large sample of
unselected female sibling pairs. Am. J. Hum. Genet. 2006;78(3):
480-486. DOI 10.1086/500052

Armanios M. The role of telomeres in human disease. Annu. Rev.
Genomics Hum. Genet. 2022;23:363-381. DOI 10.1146/annurevgenom-
010422-091101

Asghar M., Palinauskas V., Zaghdoudi-Allan N., Valkiūnas G., Mukhin
A., Platonova E., Färnert A., Bensch S., Hasselquist D. Parallel
telomere shortening in multiple body tissues owing to malaria
infection. Proc. Biol. Sci. 2016;283(1836):20161184. DOI 10.1098/
rspb.2016.1184

Astuti Y., Wardhana A., Watkins J., Wulaningsih W.; PILAR Research
Network. Cigarette smoking and telomere length: A systematic review
of 84 studies and meta-analysis. Environ. Res. 2017;158:480-
489. DOI 10.1016/j.envres.2017.06.038

Behr A.A., Liu K.Z., Liu-Fang G., Nakka P., Ramachandran S. pong:
fast analysis and visualization of latent clusters in population genetic
data. Bioinformatics. 2016;32(18):2817-2823. DOI 10.1093/
bioinformatics/btw327

Ben-Shachar M.S., Lüdecke D., Makowski D. effectsize: Estimation
of effect size indices and standardized parameters. J. Open Source
Softw. 2020;5(56):2815. DOI 10.21105/joss.02815

Blackburn E.H., Epel E.S., Lin J. Human telomere biology: A contributory
and interactive factor in aging, disease risks, and protection.
Science. 2015;350(6265):1193-1198. DOI 10.1126/science.aab3389

Broer L., Codd V., Nyholt D.R., Deelen J., Mangino M., Willemsen G.,
Albrecht E., … Vink J.M., Spector T.D., Slagboom P.E., Martin
N.G., Samani N.J., van Duijn C.M., Boomsma D.I. Meta-analysis
of telomere length in 19,713 subjects reveals high heritability,
stronger maternal inheritance and a paternal age effect. Eur. J. Hum.
Genet. 2013;21(10):1163-1168. DOI 10.1038/ejhg.2012.303

Brown D.E., Dechow C.D., Liu W.S., Harvatine K.J., Ott T.L. Hot
topic: association of telomere length with age, herd, and culling
in lactating Holsteins. J. Dairy Sci. 2012;95(11):6384-6387. DOI
10.3168/jds.2012-5593

Burraco P., Hernandez-Gonzalez M., Metcalfe N.B., Monaghan P.
Ageing
across the great divide: tissue transformation, organismal
growth and temperature shape telomere dynamics through the metamorphic
transition. Proc. Biol. Sci. 2023;290(1992):20222448. DOI
10.1098/rspb.2022.2448

Carrillo A.E., Flouris A.D. Caloric restriction and longevity: effects of
reduced body temperature. Ageing Res. Rev. 2011;10(1):153-162.
DOI 10.1016/j.arr.2010.10.001

Chakravarti D., LaBella K.A., DePinho R.A. Telomeres: history, health,
and hallmarks of aging. Cell. 2021;184(2):306-322. DOI 10.1016/
j.cell.2020.12.028

Chatelain M., Drobniak S.M., Szulkin M. The association between
stressors and telomeres in non-human vertebrates: a meta-analysis.
Ecol. Lett. 2020;23(2):381-398. DOI 10.1111/ele.13426

Chik H.Y.J., Sparks A.M., Schroeder J., Dugdale H.L. A meta-analysis
on the heritability of vertebrate telomere length. J. Evol. Biol.
2022;35(10):1283-1295. DOI 10.1111/jeb.14071

Conti B., Sanchez-Alavez M., Winsky-Sommerer R., Morale M.C., Lucero
J., Brownell S., Fabre V., Huitron-Resendiz S., Henriksen S.,
Zorrilla E.P., de Lecea L., Bartfai T. Transgenic mice with a reduced
core body temperature have an increased life span. Science. 2006;
314(5800):825-828. DOI 10.1126/science.1132191

Cook D.E., Zdraljevic S., Tanny R.E., Seo B., Riccardi D.D., Noble
L.M., Rockman M.V., Alkema M.J., Braendle C., Kammenga J.E.,
Wang J., Kruglyak L., Félix M.A., Lee J., Andersen E.C. The genetic
basis of natural variation in Caenorhabditis elegans telomere
length. Genetics. 2016;204(1):371-383. DOI 10.1534/genetics.116.
191148

Crocco P., De Rango F., Dato S., Rose G., Passarino G. Telomere length
as a function of age at population level parallels human survival
curves. Aging (Albany NY ). 2021;13(1):204-218. DOI 10.18632/
aging.202498

de Lange T. Shelterin-mediated telomere protection. Annu. Rev. Genet.
2018;52:223-247. DOI 10.1146/annurev-genet-032918-021921

Ding Z., Mangino M., Aviv A., Spector T., Durbin R. Estimating telomere
length from whole genome sequence data. Nucleic Acids Res.
2014;42(9):e75. DOI 10.1093/nar/gku181

Dmitriev N.G., Ernst L.K. (Eds.). Animal Genetics Resources of the
USSR. Rome: Food and Agriculture Organization of the United Nations,
1989

Dugdale H.L., Richardson D.S. Heritability of telomere variation: it is
all about the environment! Philos. Trans. R. Soc. Lond. B Biol. Sci.
2018;373(1741):20160450. DOI 10.1098/rstb.2016.0450

Dunin I.M., Dankvert A.G. (Eds.). Breeds and Types of Farm Animals
in the Russian Federation. Moscow: All-Russia Research Institute of
Animal Breeding, 2013 (in Russian)

Fick L.J., Fick G.H., Li Z., Cao E., Bao B., Heffelfinger D., Parker
H.G., Ostrander E.A., Riabowol K. Telomere length correlates
with life span of dog breeds. Cell Rep. 2012;2(6):1530-1536. DOI
10.1016/j.celrep.2012.11.021

Foley N.M., Petit E.J., Brazier T., Finarelli J.A., Hughes G.M., Touzalin
F., Puechmaille S.J., Teeling E.C. Drivers of longitudinal telomere
dynamics in a long-lived bat species, Myotis myotis. Mol. Ecol.
2020;29(16):2963-2977. DOI 10.1111/mec.15395

Friesen C.R., Wapstra E., Olsson M. Of telomeres and temperature:
Measuring thermal effects on telomeres in ectothermic animals. Mol.
Ecol. 2022;31(23):6069-6086. DOI 10.1111/mec.16154

Fulbert C., Gaude C., Sulpice E., Chabardès S., Ratel D. Moderate hypothermia
inhibits both proliferation and migration of human glioblastoma
cells. J. Neurooncol. 2019;144(3):489-499. DOI 10.1007/
s11060-019-03263-3

Galigniana N.M., Charó N.L., Uranga R., Cabanillas A.M., Piwien-Pilipuk G. Oxidative stress induces transcription of telomeric repeatcontaining
RNA (TERRA) by engaging PKA signaling and cytoskeleton
dynamics. Biochim. Biophys. Acta Mol. Cell. Res. 2020;
1867(4):118643. DOI 10.1016/j.bbamcr.2020.118643

Grandl F., Furger M., Kreuzer M., Zehetmeier M. Impact of longevity
on greenhouse gas emissions and profitability of individual dairy
cows analysed with different system boundaries. Animal. 2019;
13(1):198-208. DOI 10.1017/S175173111800112X

Herborn K.A., Heidinger B.J., Boner W., Noguera J.C., Adam A.,
Daunt F., Monaghan P. Stress exposure in early post-natal life reduces
telomere length: an experimental demonstration in a long-lived
seabird. Proc. Biol. Sci. 2014;281(1782):20133151. DOI 10.1098/
rspb.2013.3151

Hothorn T., Bretz F., Westfall P. Simultaneous inference in general
parametric models. Biom. J. 2008;50(3):346-363. DOI 10.1002/
bimj.20081042

Hu H., Mu T., Ma Y., Wang X., Ma Y. Analysis of longevity traits in
Holstein cattle: A review. Front. Genet. 2021;12:695543. DOI
10.3389/fgene.2021.695543

Iannuzzi A., Albarella S., Parma P., Galdiero G., D’Anza E., Pistucci
R., Peretti V., Ciotola F. Characterization of telomere length in
Agerolese cattle breed, correlating blood and milk samples. Anim.
Genet. 2022;53(5):676-679. DOI 10.1111/age.13227

Igoshin A.V., Yudin N.S., Romashov G.A., Larkin D.M. A multibreed
genome-wide association study for cattle leukocyte telomere
length. Genes (Basel). 2023;14(8):1596. DOI 10.3390/
genes14081596

Ilska-Warner J.J., Psifidi A., Seeker L.A., Wilbourn R.V., Underwood
S.L., Fairlie J., Whitelaw B., Nussey D.H., Coffey M.P., Banos
G. The genetic architecture of bovine telomere length in early
life and association with animal fitness. Front. Genet. 2019;10:
1048. DOI 10.3389/fgene.2019.01048

Jenner L.P., Peska V., Fulnečková J., Sýkorová E. Telomeres and their
neighbors. Genes (Basel). 2022;13(9):1663. DOI 10.3390/genes130
91663

Kanagawa T., Fukuda H., Tsubouchi H., Komoto Y., Hayashi S., Fukui
O., Shimoya K., Murata Y. A decrease of cell proliferation by
hypothermia in the hippocampus of the neonatal rat. Brain Res.
2006;1111(1):36-40. DOI 10.1016/j.brainres.2006.06.112

Kayumov F.G., Chernomyrdin V.N., Mayevskaya L.A., Surundaeva
L.G., Polskikh S.S. The use of Kalmyk cattle on animal breeding
farms in Russia. Izv. Orenbg. State Agrar. Univ. 2014;5(49):116-119
(in Russian)

Kordowitzki P., Merle R., Hass P.-K., Plendl J., Rieger J., Kaessmeyer
S. Influence of age and breed on bovine ovarian capillary
blood supply, ovarian mitochondria and telomere length. Cells.
2021;10(10):2661. DOI 10.3390/cells10102661

Lantz B. The impact of sample non-normality on ANOVA and alternative
methods. Br. J. Math. Stat. Psychol. 2013;66(2):224-244. DOI
10.1111/j.2044-8317.2012.02047.x

Laubenthal L., Hoelker M., Frahm J., Dänicke S., Gerlach K., Südekum
K.-H., Sauerwein H., Häussler S. Short communication: Telomere
lengths in different tissues of dairy cows during early and late
lactation. J. Dairy Sci. 2016;99(6):4881-4885. DOI 10.3168/jds.
2015-10095

Law E., Girgis A., Lambert S., Sylvie L., Levesque J., Pickett H. Telomeres
and stress: promising avenues for research in psycho-oncology.
Asia-Pacific J. Oncol. Nurs. 2016;3(2):137-147. DOI 10.4103/
2347-5625.182931

Leung C.W., Laraia B.A., Needham B.L., Rehkopf D.H., Adler N.E.,
Lin J., Blackburn E.H., Epel E.S. Soda and cell aging: associations
between sugar-sweetened beverage consumption and leukocyte telomere
length in healthy adults from the National Health and Nutrition
Examination Surveys. Am. J. Public Health. 2014;104(12):2425-
2431. DOI 10.2105/AJPH.2014.302151

Lhasaranov B. Pasture animal husbandry in Eastern Siberia. Biomed.
J. Sci. Tech. Res. 2020;31(3):24160-24163. DOI 10.26717/BJSTR.
2020.31.005094

Lin J., Epel E. Stress and telomere shortening: Insights from cellular
mechanisms. Ageing Res. Rev. 2022;73:101507. DOI 10.1016/j.arr.
2021.101507

Liu J., Wang L., Wang Z., Liu J.-P. Roles of telomere biology in cell
senescence, replicative and chronological ageing. Cells. 2019;8(1):
54. DOI 10.3390/cells8010054

López-Otín C., Blasco M.A., Partridge L., Serrano M., Kroemer G.
Hallmarks of aging: An expanding universe. Cell. 2023;186(2):243-
278. DOI 10.1016/j.cell.2022.11.001

Lynch S.M., Peek M.K., Mitra N., Ravichandran K., Branas C., Spangler
E., Zhou W., Paskett E.D., Gehlert S., DeGraffinreid C., Rebbeck
T.R., Riethman H. Race, ethnicity, psychosocial factors, and
telomere length in a multicenter setting. PLoS One. 2016;11(1):
e0146723. DOI 10.1371/journal.pone.0146723

Manning E.L., Crossland J., Dewey M.J., Van Zant G. Influences of inbreeding
and genetics on telomere length in mice. Mamm. Genome.
2002;13(5):234-238. DOI 10.1007/s003350020027

Martens D.S., Plusquin M., Cox B., Nawrot T.S. Early biological aging
and fetal exposure to high and low ambient temperature: A birth
cohort study. Environ. Health Perspect. 2019;127(11):117001. DOI
10.1289/EHP5153

McLennan D., Armstrong J.D., Stewart D.C., Mckelvey S., Boner W.,
Monaghan P., Metcalfe N.B. Telomere elongation during early development
is independent of environmental temperatures in Atlantic
salmon. J. Exp. Biol. 2018;221(Pt. 11):jeb178616. DOI 10.1242/
jeb.178616

Meesters M., Van Eetvelde M., Martens D.S., Nawrot T.S., Dewulf M.,
Govaere J., Opsomer G. Prenatal environment impacts telomere
length in newborn dairy heifers. Sci. Rep. 2023;13(1):4672. DOI
10.1038/s41598-023-31943-8

Miyashita N., Shiga K., Yonai M., Kaneyama K., Kobayashi S., Kojima
T., Goto Y., Kishi M., Aso H., Suzuki T., Sakaguchi M., Nagai T.
Remarkable differences in telomere lengths among cloned cattle derived
from different cell types. Biol. Reprod. 2002;66(6):1649-1655.
DOI 10.1095/biolreprod66.6.1649

Mizutani Y., Niizuma Y., Yoda K. How do growth and sibling competition
affect telomere dynamics in the first month of life of long-lived
seabird? PLoS One. 2016;11(11):e0167261. DOI 10.1371/journal.
pone.0167261

Monaghan P., Ozanne S.E. Somatic growth and telomere dynamics in
vertebrates: relationships, mechanisms and consequences. Philos.
Trans. R. Soc. London Ser. B. Biol. Sci. 2018;373(1741):20160446.
DOI 10.1098/rstb.2016.0446

Nowack J., Tarmann I., Hoelzl F., Smith S., Giroud S., Ruf T. Always
a price to pay: hibernation at low temperatures comes with a
trade-off between energy savings and telomere damage. Biol. Lett.
2019;15(10):20190466. DOI 10.1098/rsbl.2019.0466

O’Daniel S.E., Kochan K.J., Long C.R., Riley D.G., Randel R.D.,
Welsh T.H.J. Comparison of telomere length in age-matched primiparous
and multiparous Brahman cows. Animals (Basel). 2023;
13(14):2325. DOI 10.3390/ani13142325

Pinese M., Lacaze P., Rath E.M., Stone A., Brion M.-J., Ameur A.,
Nagpal S., … Kaplan W., Gibson G., Gyllensten U., Cairns M.J.,
McNamara M., Dinger M.E., Thomas D.M. The Medical Genome
Reference Bank contains whole genome and phenotype data of
2570 healthy elderly. Nat. Commun. 2020;11(1):435. DOI 10.1038/
s41467-019-14079-0

Purcell S., Neale B., Todd-Brown K., Thomas L., Ferreira M.A.R.,
Bender D., Maller J., Sklar P., de Bakker P.I.W., Daly M.J., Sham P.C.
PLINK: a tool set for whole-genome association and populationbased
linkage analyses. Am. J. Hum. Genet. 2007;81(3):559-575.
DOI 10.1086/519795

Rafie N., Golpour Hamedani S., Barak F., Safavi S.M., Miraghajani M.
Dietary patterns, food groups and telomere length: a systematic review
of current studies. Eur. J. Clin. Nutr. 2017;71(2):151-158. DOI
10.1038/ejcn.2016.149

Raj A., Stephens M., Pritchard J.K. fastSTRUCTURE: variational
inference of population structure in large SNP data sets. Genetics.
2014;197(2):573-589. DOI 10.1534/genetics.114.164350

Reichert S., Stier A., Zahn S., Arrivé M., Bize P., Massemin S., Criscuolo
F. Increased brood size leads to persistent eroded telomeres.
Front. Ecol. Evol. 2014;2:9. DOI 10.3389/fevo.2014.00009

Ribas-Maynou J., Llavanera M., Mateo-Otero Y., Ruiz N., Muiño R.,
Bonet S., Yeste M. Telomere length in bovine sperm is related to
the production of reactive oxygen species, but not to reproductive
performance. Theriogenology. 2022;189:290-300. DOI 10.1016/
j.theriogenology.2022.06.025

Rossiello F., Jurk D., Passos J.F., d’Adda di Fagagna F. Telomere dysfunction
in ageing and age-related diseases. Nat. Cell Biol. 2022;
24(2):135-147. DOI 10.1038/s41556-022-00842-x

Schrumpfová P.P., Fajkus J. Composition and function of telomerase-A
polymerase associated with the origin of eukaryotes. Biomolecules.
2020;10(10):1425. DOI 10.3390/biom10101425

Seeker L.A., Ilska J.J., Psifidi A., Wilbourn R.V., Underwood S.L.,
Fairlie J., Holland R., Froy H., Salvo-Chirnside E., Bagnall A.,
Whitelaw B., Coffey M.P., Nussey D.H., Banos G. Bovine telomere
dynamics and the association between telomere length and productive
lifespan. Sci. Rep. 2018a;8(1):12748. DOI 10.1038/s41598-018-
31185-z

Seeker L.A., Ilska J.J., Psifidi A., Wilbourn R.V., Underwood S.L.,
Fairlie J., Holland R., Froy H., Bagnall A., Whitelaw B., Coffey M.,
Nussey D.H., Banos G. Longitudinal changes in telomere length
and associated genetic parameters in dairy cattle analysed using
random regression models. PLoS One. 2018b;13(2):e0192864. DOI
10.1371/journal.pone.0192864

Seeker L.A., Underwood S.L., Wilbourn R.V., Dorrens J., Froy H., Holland
R., Ilska J.J., Psifidi A., Bagnall A., Whitelaw B., Coffey M.,
Banos G., Nussey D.H. Telomere attrition rates are associated with
weather conditions and predict productive lifespan in dairy cattle.
Sci. Rep. 2021;11(1):5589. DOI 10.1038/s41598-021-84984-2

Sleptsov I.I., Machakhtyrova V.A., Ivanova N.P. Clinical and physiological
indicators of the Kalmyk cattle breed in Yakutia conditions.
Bull. Kurgan State Agric. Acad. 2019;4(32):44-46 (in Russian)

Szczotka M., Kocki J., Iwan E., Pluta A. Determination of telomere
length and telomerase activity in cattle infected with bovine leukaemia
virus (BLV). Pol. J. Vet. Sci. 2019;22(2):391-403. DOI
10.24425/pjvs.2019.129299

Taub M.A., Conomos M.P., Keener R., Iyer K.R., Weinstock J.S.,
Yanek L.R., Lane J., … de Andrade M., Correa A., Chen Y.I., Boerwinkle
E., Barnes K.C., Ashley-Koch A.E., Arnett D.K.; NHLBI
Trans-Omics for Precision Medicine (TOPMed) Consortium;
TOPMed Hematology and Hemostasis Working Group; TOPMed
Structural Variation Working Group; Laurie C.C., Abecasis G.,
Nickerson D.A., Wilson J.G., Rich S.S., Levy D., Ruczinski I.,
Aviv A., Blackwell T.W., Thornton T., O’Connell J., Cox N.J.,
Perry J.A., Armanios M., Battle A., Pankratz N., Reiner A.P., Mathias
R.A. Genetic determinants of telomere length from 109,122 ancestrally
diverse whole-genome sequences in TOPMed. Cell Genom.
2022;2(1):100084. DOI 10.1016/j.xgen.2021.100084

Tilesi F., Di Domenico E.G., Pariset L., Bosco L., Willems D., Valentini
A., Ascenzioni F. Telomere length diversity in cattle breeds. Diversity.
2010;2(9):1118-1129. DOI 10.3390/d2091118

Waalen J., Buxbaum J.N. Is older colder or colder older? The association
of age with body temperature in 18,630 individuals. J. Gerontol.
A Biol. Sci. Med. Sci. 2011;66(5):487-492. DOI 10.1093/gerona/
glr001

Whittemore K., Vera E., Martínez-Nevado E., Sanpera C., Blasco M.A.
Telomere shortening rate predicts species life span. Proc. Natl.
Acad. Sci. USA. 2019;116(30):15122-15127. DOI 10.1073/pnas.
1902452116

Wilbourn R.V., Moatt J.P., Froy H., Walling C.A., Nussey D.H.,
Boonekamp J.J. The relationship between telomere length and mortality
risk in non-model vertebrate systems: a meta-analysis. Philos.
Trans. R. Soc. London Ser. B. Biol. Sci. 2018;373(1741):20160447.
DOI 10.1098/rstb.2016.0447

Zepner L., Karrasch P., Wiemann F., Bernard L. ClimateCharts.net – an
interactive climate analysis web platform. Int. J. Digit. Earth. 2021;
14(3):338-356. DOI 10.1080/17538947.2020.1829112

Zhang D., Newton C.A., Wang B., Povysil G., Noth I., Martinez F.J.,
Raghu G., Goldstein D., Garcia C.K. Utility of whole genome sequencing
in assessing risk and clinically relevant outcomes for pulmonary
fibrosis. Eur. Respir. J. 2022;60(6):2200577. DOI 10.1183/
13993003.00577-2022

Zhang H., Liu A., Wang Y., Luo H., Yan X., Guo X., Li X., Liu L.,
Su G. Genetic parameters and genome-wide association studies of
eight longevity traits representing either full or partial lifespan in
Chinese Holsteins. Front. Genet. 2021;12:634986. DOI 10.3389/
fgene.2021.634986

Zhang X., Lin S., Funk W.E., Hou L. Environmental and occupational
exposure to chemicals and telomere length in human studies.
Occup. Environ. Med. 2013;70(10):743-749. DOI 10.1136/
oemed-2012-101350

Zhang Y., Wu Y., Mao P., Li F., Han X., Zhang Y., Jiang S., Chen Y.,
Huang J., Liu D., Zhao Y., Ma W., Songyang Z. Cold-inducible RNAbinding
protein CIRP/hnRNP A18 regulates telomerase activity in
a temperature-dependent manner. Nucleic Acids Res. 2016;44(2):
761-775. DOI 10.1093/nar/gkv1465

Zhao Z., Cao J., Niu C., Bao M., Xu J., Huo D., Liao S., Liu W., Speakman
J.R. Body temperature is a more important modulator of lifespan
than metabolic rate in two small mammals. Nat. Metab. 2022;
4(3):320-326. DOI 10.1038/s42255-022-00545-5

